# Insight into the Influence of Cultivar Type, Cultivation Year, and Site on the Lignans and Related Phenolic Profiles, and the Health-Promoting Antioxidant Potential of Flax (*Linum usitatissimum* L.) Seeds

**DOI:** 10.3390/molecules23102636

**Published:** 2018-10-14

**Authors:** Laurine Garros, Samantha Drouet, Cyrielle Corbin, Cédric Decourtil, Thibaud Fidel, Julie Lebas de Lacour, Emilie A. Leclerc, Sullivan Renouard, Duangjai Tungmunnithum, Joël Doussot, Bilal Haider Abassi, Benoit Maunit, Éric Lainé, Ophélie Fliniaux, François Mesnard, Christophe Hano

**Affiliations:** 1 Laboratoire de Biologie des Ligneux et des Grandes Cultures (LBLGC) EA1207 INRA USC1328, Plant LIGNANS Team, Université d’Orléans, 28000 Chartres, France; laurine.garros@univ-orleans.fr (L.G.); samantha.drouet@univ-orleans.fr (S.D.); cyrielle.corbin@univ-orleans.fr (C.C.); cedric.decourtil@univ-orleans.fr (C.D.); thibaud.fidel@univ-orleans.fr (T.F.); julie.lebas-de-lacour@univ-orleans.fr (J.L.d.L.); emilie.leclerc@univ-orleans.fr (E.A.L.); sullivan.renouard@univ-orleans.fr (S.R.); duangjai.tun@mahidol.ac.th (D.T.); joel.doussot@lecnam.net (J.D.); bhabbasi@qau.edu.pk (B.H.A.); eric.laine@univ-orleans.fr (É.L.); 2 COSM’ACTIFS, Bioactifs et Cosmétiques, CNRS GDR3711, 45067 Orléans Cedex 2, France; benoit.maunit@univ-orleans.fr; 3 Institut de Chimie Organique et Analytique (ICOA) UMR7311, Université d’Orléans-CNRS, 45067 Orléans CEDEX 2, France; 4 Department of Pharmaceutical Botany, Faculty of Pharmacy, Mahidol University, 447 Sri-Ayuthaya Road, Rajathevi, Bangkok 10400, Thailand; 5 Le CNAM, Ecole Sciences Industrielles et Technologies de l’Information (SITI), Chimie Alimentation Santé Environnement Risque (CASER), 75141 Paris Cedex 3, France; 6 Department of Biotechnology, Quaid-i-Azam University, 45320 Islamabad, Pakistan; 7 Biologie des Plantes et Innovation (BIOPI) EA 3900, Université de Picardie Jules Verne, 80000 Amiens, France; ophelie.fliniaux@u-picardie.fr (O.F.); francois.mesnard@u-picardie.fr (F.M.)

**Keywords:** cultivar, environment, flax, flavonol, genetic, hydroxycinnamic acid, lignan, seed

## Abstract

Flaxseeds are a functional food representing, by far, the richest natural grain source of lignans, and accumulate substantial amounts of other health beneficial phenolic compounds (i.e., flavonols, hydroxycinnamic acids). This specific accumulation pattern is related to their numerous beneficial effects on human health. However, to date, little data is available concerning the relative impact of genetic and geographic parameters on the phytochemical yield and composition. Here, the major influence of the cultivar over geographic parameters on the flaxseed phytochemical accumulation yield and composition is evidenced. The importance of genetic parameters on the lignan accumulation was further confirmed by gene expression analysis monitored by RT-qPCR. The corresponding antioxidant activity of these flaxseed extracts was evaluated, both in vitro, using ferric reducing antioxidant power (FRAP), oxygen radical absorbance capacity (ORAC), and iron chelating assays, as well as in vivo, by monitoring the impact of UV-induced oxidative stress on the lipid membrane peroxidation of yeast cells. Our results, both the in vitro and in vivo studies, confirm that flaxseed extracts are an effective protector against oxidative stress. The results point out that secoisolariciresinol diglucoside, caffeic acid glucoside, and *p*-coumaric acid glucoside are the main contributors to the antioxidant capacity. Considering the health benefits of these compounds, the present study demonstrates that the flaxseed cultivar type could greatly influence the phytochemical intakes and, therefore, the associated biological activities. We recommend that this crucial parameter be considered in epidemiological studies dealing with flaxseeds.

## 1. Introduction

**Figure 1:**
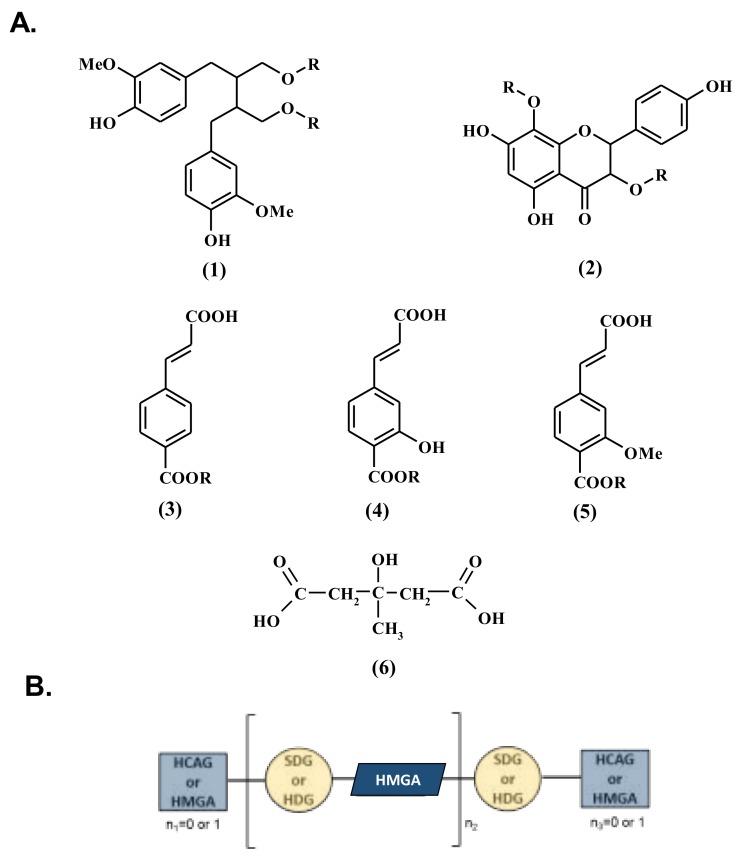
Structure of phenolic compounds involved in the lignan macromolecular complex. (**A**) Structure of the complex components: (**1**) secoisolariciresinol (R = H), or secoisolariciresinol diglucoside (SDG, R = *β*-d-glucose), (**2**) herbacetin (R = H) or herbacetin diglucoside (HDG, R = *β*-d-glucose), (**3**) *p*-coumaric acid (R = H), or *p*-coumaric acid glucoside (CouG, R = *β*-d-glucose), (**4**) caffeic acid (R = H) or caffeic acid glucoside (CafG, R = *β*-d-glucose), (**5**) ferulic acid (R = H) or ferulic acid glucoside (FerG, R = *β*-d-glucose), (**6**) hydroxymethylglutaric acid (HMGA). (**B**) Schematic representation of lignan macromolecule, where a unit of SDG or HDG is ester-linked to another unit, thanks to HMGA, which can be replaced by one hydroxycinnamic acid glucoside (HCAG) unit (CouG, CafG, or FerG) in terminal position of the chain.

The consumption of fruit, vegetables, and grains has been associated with lower risks of chronic and degeneration diseases [1]. Considering their numerous beneficial effects on human health, during the last decades, there has been an increasing interest in their uses, and flaxseeds are, therefore, considered as functional food [2]. Flaxseeds are the richest natural grain source of lignan and accumulate a substantial amount of other phenolic compounds (e.g., flavonols, hydroxycinnamic acids). In flaxseed, the foremost part of these phytochemicals is accumulated under the form of a macromolecular complex (also known as lignan macromolecule) composed of the lignan secoisolariciresinol diglucoside (SDG, Figure 1A) as the main component, and of flavonol herbacetin diglucoside (HDG, Figure 1A), as well as hydroxycinnamic acid derivatives: *p*-coumaric acid glucoside (CouG, Figure 1A), caffeic acid glucoside (CafG, Figure 1A), and ferulic acid glucoside (FerG, Figure 1A), ester-linked together to hydroxymethylglutaryl spacers (Figure 1B) [3,4].

The beneficial effects of lignans on human health are well recognized [5,6]. Particularly, the chemopreventive actions of SDG toward cancer, diabetes mellitus, and cardiovascular diseases have been largely described [5,7,8]. The pharmacological activity of this compound is thought to be due to its high antioxidant capacity [9,10,11] and to its phytoestrogenic activity [12]. Flavonols and hydroxycinnamic acids, the other constituents of the flaxseed lignan macromolecule, also display a wide range of health-promoting effects. The favorable actions on cardiovascular health of vegetable-rich diets have been ascribed to flavonols, and hydroxycinnamic acids have revealed powerful antioxidant properties and might be of particular interest for dermatologic applications [13,14].

Although both in vivo and in vitro data are globally in favor of a chemopreventive effect of lignans, epidemiological studies are much less conclusive, and the mechanism by which phytoestrogenic lignans prevent cancers still remains unclear [7] and requires further elucidation. This could be explained by the fact that our current knowledge concerning the genetic and environmental factors affecting productivity and yield stability of these phenolic compounds, in flaxseeds, remains partial, and little is known about the variation in antioxidant capacities of different flaxseed cultivars. Moreover, no study has put efforts toward linking the lignan content of different cultivars and the expression of genes involved in their biosynthetic pathway.

Herein we present a complete dataset concerning the relative impact of cultivar, edaphic, and climatic parameters on productivity of the main constituents of the lignan macromolecule of flaxseeds, in relation to their antioxidant capacities determined using both in vitro and in vivo systems. Such data could be useful to predict, more precisely, the accumulation and, therefore, the nutritional intakes of these compounds, with health benefits for pharmaceutical, nutraceutical, and/or cosmetic applications.

## 2. Materials and Methods

### 2.1. Chemicals

All chemicals were of analytical grade quality and purchased from Thermo (Illkirch, France). The deionized water was produced using a milli-Q water purification system (Merck Millipore, Molsheim, France). SDG and HDG standard were purchased from LGC Standards (Molsheim, France). The hydroxycinnamic acid glucosides—*p*-coumaric acid glucoside, caffeic acid glucoside, ferulic acid glucoside—were synthesized according to Beejmohun et al. (2004) [15] and Beejmohun et al. (2006) [16]. Prior to their use for HPLC or LC-MS analysis, all solutions were filtered through 0.45 µm nylon syringe membranes (Merck Millipore, Molsheim, France). 

### 2.2. Plant Materials and Cultivation

Flax cultivars Astral, Baïkal, Baladin, Barbara, and Oliver were provided by Laboulet Semences (Airaines, France), Coopérative Linière Terre de Lin (Saint-Pierre-le-Viger, France) and Arvalis-Institut Technique du Lin (Boigneville, France). Flax was grown up to seed at the following locations in France: Eure (Gamaches-en-Vexin, GAM, 49°16′14′’N/1°37′02′’E/89 m), Somme (Airaines, AIR, 49°57′57″N/1°56′39″/70 m), and Eure-et-Loir (Chartres, CHA, 48°27′21.05″N/1°29′3.06″E/141 m). Sowings were performed on March 30th of each year, with 450 seeds per m^2^. Fields were fertilized, immediately after sowing, with 80 units of nitrogen, 60 units of potassium, and 60 units of phosphorus per hectare (Figure S1). The soils of these sites were of clay loam type balanced, well-structured with a granulometry of ca. 25% 2000–63 µm, 50% 63–2 µm, and 25% <2 µm particles, and a pH around 7.8. The final harvest took place on August 15th of each year at the same ripening stage for each cultivar. Throughout the experiments, no visible disease or insect attack occurred at either location. During the growing period, the experimental stations received 182.8 mm (year 2003), 305.0 mm (year 2004), 269.8 mm (year 2005) for GAM, 387.4 mm for AIR (year 2005), and 308.6 mm for CHA (year 2005) of rainfall over the growing period. The day temperatures at an elevation of 2 m averaged 15.72 °C (year 2003), 13.67 °C (year 2004), 13.96 °C (year 2005) for GAM, 13.48 °C for AIR (year 2005), and 14.45 °C for CHA (year 2005) over the growing period. All these meteorological characteristics are displayed in Table S1 and Figure S2.

### 2.3. Gene Expression Analysis by RT-qPCR

Total RNA was extracted from 100 mg of frozen plant material in liquid nitrogen as described by Hano et al. (2006) [17]. Expression patterns of *LuDIR5*, *LuPLR1*, and *LuUGT74S1* were analyzed using RT-qPCR, using specific primers described by Dalisay et al. (2015) [18]. For reverse transcription, 50 ng of total RNA was incubated for 60 min at 50 °C with 1× RT buffer, 0.5 mM of each dNTP, 1 μM of oligo-dT primers, 1 unit of RiboLock, and 4 units of Omniscript Reverse Transcriptase in a total volume of 20 μL (Qiagen, Hilden, Germany). qPCR was performed with a PikoReal™ Real-Time PCR System (Thermo Fisher Scientific, Villebon-sur-Yvette, France) using DyNAmo ColorFlash SYBR Green qPCR (ThermoScientific) and specific primers. Two reference genes (*CYC* and *ETIF5A*) were used for data normalization [19]. The qPCR parameters were as follows: an initial denaturation at 95 °C for 5 min, then 40 three-step cycles of 94 °C for 10 s, primer annealing at 65 °C for 10 s, and extension at 72 °C for 30 s. After 40 cycles, an additional extension step was performed at 72 °C for 90 s. The presence of a single amplicon was confirmed by the observation of a single peak in the melting curve obtained after amplification. Expression levels were calculated and normalized using 2^−ΔΔCt^ method [20]. Reactions were performed in three biological and two technical replicates.

### 2.4. Extraction, HPLC, and LC-ESI-MS Analysis

Extractions (4 biological and 2 technical replicates), quantification of compounds was carried out on a Varian liquid chromatographic system (Agilent Technology, Les Ulis, France), as well as LC-ESI-MS analyses using a Waters 2695 Alliance coupled with a single quadrupole mass spectrometer ZQ (Waters-Micromass, Manchester, UK), equipped with an electrospray ion source (ESI-MS), were performed as described in Corbin et al. (2015) [21].

### 2.5. Determination of the Ferric-Reducing Antioxidant Power (FRAP)

Ferric-reducing antioxidant power (FRAP) was measured as described by Benzie & Strain, (1996) [22] with little modification. Briefly, 10 μL of the extracted sample was mixed with 190 μL of FRAP (10 mM TPTZ; 20 mM FeCl_3_∙6H_2_O, and 300 mM acetate buffer pH 3.6; ratio 1:1:10 (v/v/v)). Incubation lasted 15 min at room temperature. Absorbance of the reaction mixture was measured at 630 nm with a BioTek ELX800 Absorbance Microplate Reader (Thermo Fisher Scientific, Villebon-sur-Yvette, France). Assays were made in triplicate and antioxidant capacity was expressed as Trolox C equivalent antioxidant capacity (TAEC).

### 2.6. Determination of Oxygen Radical Absorbance Capacity (ORAC)

Oxygen radical absorbance capacity (ORAC) assay was performed as described by Prior et al. (2003) [23]. Briefly, 10 μL of the extracted sample was mixed with 190 μL of fluorescein (0.96 µM) in 75 mM phosphate buffer pH 7.4, and incubated for at least 20 minutes at 37 °C with intermittent shaking. Then, 20 µL of 119.4 mM 2,2′-azobis-amidinopropane (ABAP, Sigma Aldrich, Saint-Quentin Fallavier, France) was added and the fluorescence intensity was measured every 5 min for 2.5 h at 37 °C using a fluorescence spectrophotometer (Bio-Rad, Marnes-la-Coquette, France) set with an excitation at 485 nm and emission at 535 nm. Assays were made in triplicate, and antioxidant capacity was expressed as Trolox C equivalent antioxidant capacity (TAEC).

### 2.7. Determination of the Iron-Chelating Capacity

The iron-chelating capacity was determined as described by Mladenka et al. (2011) [24]. Briefly, 10 µL of extract sample were mixed with ferrous iron at a final concentration of 50 μM in HEPES (pH 6.8) buffer and 50 µL ferrozine (5 mM aqueous solution). All experiments were performed in 96-well microplates. Each sample was measured with and without (blank) the addition of ferrozine. Absorbance was measured at 550 nm immediately after addition of ferrozine, and 5 min later with a BioTek ELX800 Absorbance Microplate Reader (Thermo Fisher Scientific, Villebon-sur-Yvette, France). Chelating activity values were expressed in µM of fixed iron.

### 2.8. Yeast Cells Cultivation and Treatments

Yeast (*Saccharomyces cerevisiae*) strain MAV203 (Invitrogen, Thermo Fisher Scientific Villebon-sur-Yvette, France) were used. Cells were grown aerobically at 30 °C in an orbital shaker (150 rpm) in complete 2.0% (w/v) glucose YPD medium (Sigma Aldrich, Saint-Quentin Fallavier, France). All extracts evaporated under nitrogen flow, dissolved in DMSO at 50 µg/mL, and added to the cells 6 h before oxidative stress induction at a final concentration of 1 mg/mL. The final concentration of DMSO applied on the cell was 1 % (v/v). For the control sample, DMSO to 0.1% of the final volume, was added. Cells were irradiated with 106.5 J/m^2^ UV-C (254 nm) under a Vilber VL-6.C filtered lamp (Thermo Fisher Scientific, Villebon-sur-Yvette, France), as described by Bisquert et al. (2018) [25], and then incubated overnight at 30 °C before membrane lipid peroxidation determination.

### 2.9. Determination of Membrane Lipid Peroxidation Using Thiobarbituric Acid-Reactive Substances (TBARS) Assay

Measurement of membrane lipid peroxide was carried out with the thiobarbituric acid (TBA; Sigma Aldrich, Saint-Quentin Fallavier, France) method described by Hano et al. (2008) [26]. Briefly, ca. 10^7^ cells were ground using a mortar and pestle in distilled water, and centrifuged at 10,000×*g* for 10 min. Supernatant fractions (75 µL) were mixed with 25 µL of 3% (w/v) SDS, 50 µL of 3% TBA (w/v) in 50 mM NaOH, and 50 µL of 23% (v/v) of HCl throughout mixing between each addition. The mixture was heated at 80 °C for 20 min. After cooling on ice, the absorbance at 532 nm (A532) was measured, and non-specific absorbance at 600 nm (A600) was subtracted.

### 2.10. Statistical Treatment of Data

All data presented in this study are the means and the standard deviations of at least three independent replicates. ANOVAs and Pearson correlations were performed using R software version 3.0.2. PCA was performed with XL-STAT2017 software (Addinsoft, Paris, France), with each parameter considered as a discrete variable; the initial dataset was then converted into principal components (PCs), and it was possible to graphically display the relationships among the considered parameters. Gene expression and SDG content were represented using MeV4 software. All statistical tests were considered significant at *p* < 0.05. 

## 3. Results and Discussion

### 3.1. Influence of Genetic Variations on the Accumulation of the Main Constituents of the Lignan Macromolecule

The flax cultivars, herein studied, showed an SDG content ranging from 8.23 to 21.85 mg/g of dry weight (DW) (Table 1). Barbara and Oliver are high SDG-producing cultivars, Baladin presents an intermediate content, whereas Astral and Baïkal are poor in SDG, as compared to the other cultivars. A similar range of variation in SDG content has been reported in a flax germplasm collection by Diederichsen and Fu (2008) [27]. Lower SDG content was reported by Zimmermann et al. (2007, 2006) [28,29] for cultivars grown in Spain and Germany. Nevertheless, it should be noted that these authors employed an extraction method based on acid hydrolysis, which is known to be potentially destructive for SDG [30], leading to a possible underestimation in the actual contents. SDG is the main component of the lignan macromolecule accumulated in flaxseed, but other compounds, such as hydroxycinnamic acid glucosides (caffeic acid glucoside (CafG), *p*-coumaric acid glucoside (CouG), and ferulic acid glucoside (FerG), as well as the flavonol herbacetin diglucoside (HDG), are also incorporated in substantial amounts in this macromolecule [4,31,32]. Here, the whole set of these compounds was assayed. In our hands, CouG contents ranged from 4.78 to 10.48 mg/g DW, and FerG content from 1.03 to 2.28 mg/g DW (Table 1). These results sound consistent with those described by Westcott and Muir (1996), Jonhson et al. (2000), and Eliasson et al. (2003) [33,34,35] for cultivars grown respectively in Canada, Denmark, and Sweden. To date, only semi-quantitative evaluation of the HDG variations in flax cultivars have been studied through NMR [36], therefore, to the best of our knowledge, the present work is the first study focusing on the quantitative variations in HDG contents in linseed cultivars. Concerning the quantitative variations in CafG, only Wang et al. (2017) [37] reported very low contents ranging from 2.40 to 8.70 µg/g DW for Chinese cultivars. Here, HDG content ranged from 0.75 to 1.18 mg/g DW, and CafG contents from 0.80 to 1.90 mg/g DW (Table 1).

**Figure 2:**
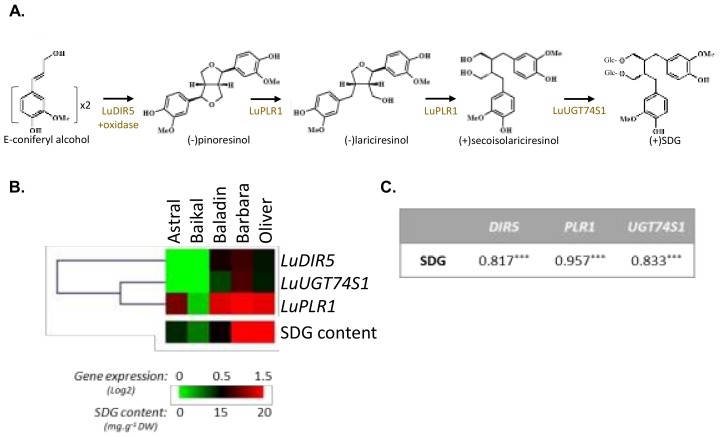
Expression profile of flax lignan biosynthetic gene and SDG accumulation in five flaxseed cultivars. (**A**) Biosynthetic pathway leading to the formation of (+)-SDG in flaxseed. (**B**) Expression of *LuDIR5*, *LuPLR1*, and *LuUGT74S1* determined by RT-qPCR (normalized with *CYC* and *ETIF5A* reference genes) visualized using MeV4 (*n* = 3) and SDG content measured by HPLC and visualized using MeV4 (*n* = 3). (**C**) Pearson correlation matrix between (+)-SDG accumulation and the corresponding biosynthetic gene expression. Significance level: * *p* < 0.05; ** *p* < 0.01; *** *p* < 0.001.

In flaxseed, the lignan biosynthesis involves the dirigent protein (*LuDIR5;* Figure 2A)-mediated stereoselective coupling of two *E*-coniferyl alcohol moieties, resulting in the formation of (−)-pinoresinol [18,38]. The two following reaction steps leading to the conversion of (−)-pinoresinol to (−)-lariciresinol, and (−)-lariciresinol to (+)-secoisolariciresinol, are catalyzed by the same bifunctional enzyme pinoresinol–lariciresinol reductase (*LuPLR1*, Figure 2A) [17,39,40]. Secoisolariciresinol is then glycosylated into SDG under the control of UDP-glycosyltransferase (*LuUGT74S1*, Figure 2A) glycosylating the C-9 and C-9’ hydroxyl positions [41,42]. SDG is stored as a 3-hydroxy-3-methylglutaryl ester-linked complex (HMG-SDG), as shown in Figure 1. Formation of the HMG–SDG ester-linked oligomers, occurs by linking hydroxylmethylglutaryl (HMG) to C-6a and C-6a’ position, via action of HMG CoA-transferase [43].

Correlation analysis between the different constituents of the flax lignan macromolecule revealed significant positive correlations between the CafG, CouG, and SDG contents, on the one hand, and between HDG and FerG, on the other hand (Table 2). This correlation was in agreement with our previous results [36]. On the contrary, significant negative correlations were noted between the SDG vs HDG and FerG yields (Table 2), which confirmed our previous observations [36]. From a metabolic point of view, *p*-coumaric acid (Figure 1A) is a branch point leading to the biosynthesis of either flavonoids or lignans [44]. Therefore, caffeic acid and *p*-coumaric acid (Figure 1A) could be considered as more direct precursors for the HDG biosynthesis, whereas ferulic acid (Figure 1A) constitutes a precursor for SDG biosynthesis. These biosynthetic links could explain, in part, the observed correlations. Studying the possible metabolic channel regulation of the carbon allocation between these two branches, during flaxseed development, could be of particular interest.

As a step forward, lignan biosynthetic gene expression analysis performed on immature flaxseed (developmental stage 2; [17]) by RT-qPCR using the 3 specific genes involved in SDG biosynthesis (*LuDIR5*, *LuPLR1*, and *LuUGT74S1*; Figure 2A,B) appeared in good agreement with the HPLC quantification (Figure 2C). High expression of *LuPLR1* was detected in high SDG-producing cultivars, Barbara and Oliver, whereas Astral and Baïkal cultivars, accumulating lower SDG content, showed a lower expression of these biosynthetic genes (Table 1). The steady state levels of the key *LuPLR1* transcripts [40], and the two other biosynthetic genes (*LuDIR5* and *LuUGT74S1*) are correlated with the SDG content measured in the corresponding mature seeds (Figure 2C), confirming the great influence of genetic parameters (i.e., the cultivar), and indicated that most of the regulation occurred at transcriptional level.

### 3.2. Influence of Geographic Parameters on the Accumulation of the Main Constituents of Lignan Macromolecule

It is well accepted that environmental conditions, such as the climate of the culture year and the location (soil conditions), could also greatly affect the accumulation of phenolic compounds, as previously observed by Oomah et al. (1996) [45] for the accumulation of total flavonoids in flaxseeds. Here, three different locations have been selected to provide access to the potential influence of edaphic condition on lignan accumulation in flaxseed. Bordered by four different seas, three mountain ranges, and the edge of the central European lowlands, France is known to be a country with very diverse climatic conditions, resulting in very different weather patterns. Here, the three selected experimental sites are representative of the major flax-growing areas in France, i.e., the western part, and the present contrasting weather patterns. The CHA site is characterized by the highest temperatures and the lowest rainfall during the seed maturation phase. On the contrary, AIR location presents the lowest temperatures and the highest rainfall observed during the same period. The last location, GAM, is considered as an intermediate in terms of climate. The impact of these different conditions, on the composition and amount of the main constituents of the lignan macromolecule accumulated in the seed of the five selected cultivars, are presented in Table 1. Analysis of the variance revealed that cultivar was the main contributor for the observed variability (cultivars, C, Table 1). Edaphic factor (location L, Table 1) has no significant effect on the accumulation of these phytochemicals, whereas significant interactions with genetic factor were noted, but evidenced the prominent effect of genetic background at a particular location according to *F* values (Table 1).

Nonetheless, the location constitutes a complex variable, differing by both climatic and edaphic parameters, thus, to evaluate the sole contribution of climate, we decided to compare flaxseeds grown at the same site, GAM (i.e., the same edaphic parameters) but in different cultivation years (i.e., different climatic parameters). Here, we chose to consider three consecutive years with very contrasting weather patterns and, for this reason, the 2003–2005 period was selected. Indeed, it must be noted that the summer of 2003 was the hottest and driest in recent decades, and must be regarded as extremely unusual. The 2003–2005 period was also the warmest period recorded in France since 1950, whereas the low rainfall observed from June 2004 to December 2005 led to a dramatic soil water deficit for 2005, with a soil humidity index close to 0.25 for GAM region (considering that a soil humidity index of 1 is for water-saturated soil whereas 0 is for water-depleted soil; see Table S1, Figure S2 for complete meteorological condition descriptions). As flax is known to be a water-demanding crop during its flowering period (i.e., June), we therefore decided to evaluate how these climate changes, leading to water deficiency during this period, have affected the flaxseed metabolism. The results are reported in Table 1, and the analysis of the variance evidenced the genetic background (cultivars, C, Table 1) as the sole significant factor influencing the SDG, FerG, CouG, and CafG content (Table 1). The climatic parameters considered here (cultivation year, Y, Table 1) did not influence the accumulation of any molecules in the analyzed cultivars, whereas significant interaction between genetic and climatic parameters (C × Y, Table 1) was noted, but with lower *F* values as compared to genetic parameter alone (C, Table 1), indicating that the main contributions have to be attributed to this latter parameter. Our results are in good agreement with the results of Saastamoinen et al. (2013) [46], who also reported a lower impact of the cultivation year compared to the cultivar parameter on SDG accumulation. On the contrary, Wescott et al. (2002) [47] reported that the cultivation year could also influence SDG yield. This apparently contradictory result can be due to the complexity of the climatic variable, that could also be influenced by the nature of the soil considered (edaphic parameters). The nature of the soil could greatly affect the influence of the drought period as its ability to retain water greatly relied on its composition and granulometry. Indeed, a high soil ability to retain water could alleviate the effect of temporary drought, and differences in this feature could explain such apparent discrepancies, moreover, the rainfall regime differs between Scandinavian and Canadian summers, making it more probable that drought occurs during the latter.

**Figure 3:**
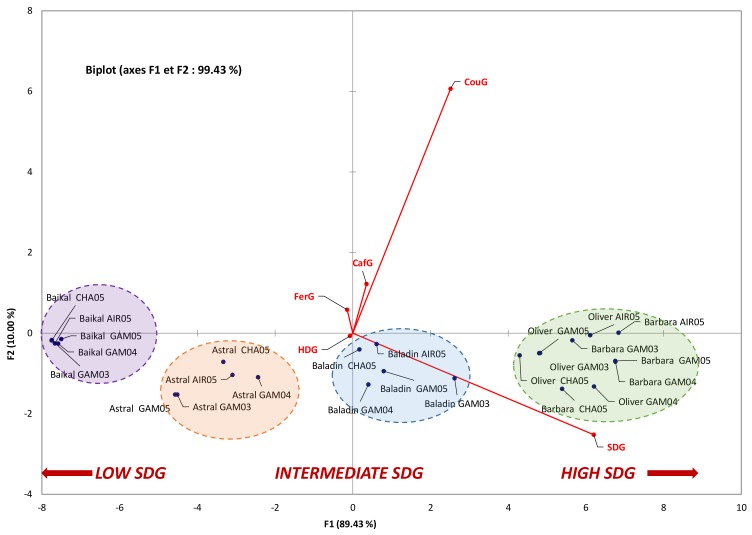
Correlation circle for principal component analysis. The SDG, HDG, CafG, CouG, and FerG contents for 5 cultivars (Astral, Baïkal, Baladin, Barbara, and Oliver) growing at 3 different locations (GAM, AIR, and CHA) and over 3 different years (03 (2003), 04 (2004), or 05 (2005)) were submitted for analysis by the PCA algorithm in Excel-XLSTAT software, using the Pearson correlation matrix (at a significance level of *p* < 0.05).

All these phytochemical profiles were subjected to principal component analysis. The resulting biplot representation accounts for 99.43% (F1 + F2) of the initial variability of the data (Figure 3). Discrimination occurs mainly in the first dimension, and SDG content was the main contributor for this F1 axis that explains 89.43% of the initial variability. The concentrations of hydroxycinnamic acid glucosides (particularly CouG) were the main contributors for the second dimension (F2 axis), accounting for only 10% of the initial variability (Figure 3). PCA showed a significant grouping of samples as a function of their SDG content. Using this analysis, the different cultivars could also be easily discriminated. This PCA confirmed the prominence of the genetic background over the environmental (edaphic and climatic) factors studied here.

### 3.3. Evaluation and Comparison of In Vitro and In Vivo Antioxidant Capacities

To evaluate the influence of genetic and edaphic variables on the health benefit potential of these flaxseeds, the antioxidant capacity of the corresponding extracts was then evaluated using both in vitro and in vivo assays. On the basis of the chemical reaction involved, the major antioxidant capacity assays can be roughly divided into two categories: i) hydrogen atom transfer (HAT) reaction-based assay, such as ORAC assay, or ii) electron transfer (ET) reaction-based assay, such as FRAP assay (Table 3).

In our hands, the antioxidant capacity of our flaxseed extracts revealed by these two different assays ranged from 217.35 (Baladin, AIR_05) to 355.75 (Oliver, GAM_05) µM of Trolox C equivalent antioxidant capacity (TEAC) using FRAP assay, and from 269.97 (Baïkal, GAM_04) to 375.76 (Oliver, GAM_05) µM TEAC using ORAC assay (Table 3). The antioxidant capacity of polyphenolic compounds, such as lignans, has been previously attributed to their capacity for HAT, from their OH groups to the free radicals [48]. However, the radical scavenging capacity of these extracts occurring through an ET-based mechanism cannot be excluded, according to the high antioxidant values calculated from the FRAP assay (Table 3). Here these two in vitro antioxidant assays were significantly correlated with the presence of SDG, CouG and CafG (Table 2).

Besides these two mechanisms involved in the scavenging of reactive oxygen species, transient metal ion chelation is also considered as an antioxidant mechanism, since the Fenton reaction, responsible for the hydroxyl radical formation and, subsequently, radical chain reaction propagation, could be inhibited through this chelating mechanism [49,50]. Here, we evidenced that flaxseed extracts displayed an efficient iron (Fe^2+^)-chelating activity, ranging from 7.18 µM (Baïkal, GAM_03) to 14.72 µM (Oliver, GAM_04) of fixed iron (Table 3), that could also contribute to their antioxidant activity, largely described in the literature [9,10]. In good agreement with the recent rationalization of the iron-chelating capacity of SDG and its aglycone form secoisolariciresinol, high SDG quantities associated with elevated contents of CouG and CafG, appeared to significantly contribute to the development of a high iron-chelating capacity of the corresponding flaxseed extracts (Table 2).

It is necessary to emphasize that the assays described herein are strictly predictive results based on the chemical reaction in vitro, however, they not necessary bear a great similarity to biological systems. The validity of these data has to be, therefore, considered as limited to a strict chemical sense with context interpretation. For this reason, in order to better reflect the in vivo situation, the antioxidant activity of these extracts was further investigated for their capacity to inhibit membrane lipid peroxidation induced by UV-C in yeast cells. Yeast cells have been proven to be an excellent model to evaluate in vivo antioxidant capacity in a cellular oxidative stress context [51]. Indeed, baker’s yeast (*Saccharomyces cerevisiae*) is an attractive and reliable model. This organism is a true eukaryote, and the mechanisms of defense and adaptation to oxidative stress are well understood [25,52]. The in vivo anti-lipoperoxidation activity (inhibition of malondialdehyde (MDA) formation), determined using the TBARS assay, ranged from 21.68% (Baïkal, GAM_04) to 47.02% (Oliver, AIR_05) (Table 3). Interestingly, a strong and significant correlation was observed between this cellular antioxidant capacity and the SDG (PCC = 0.867), CouG (PCC = 0.806), and CafG (PCC = 0.721) contents (Table 2). However, we can note that, since the contents of SDG, CouG, and CafG are highly correlated, these parameters are not independent, and it is, therefore, difficult to definitely judge their respective contribution to this biological activity (cellular antioxidant capacity) by single correlation analysis. In yeast models, a similar protective effect against oxidative stress was previously observed on yeast cells treated with thiamine [52] and melatonin [25]. To the best of our knowledge, this the first time that this system is applied to characterize a flax extract. Our results are in agreement with those obtained using in vitro assays, and highlighted the great in vivo antioxidant potential of flaxseed extracts as already proposed by Wang et al. (2017) [37], using another cellular antioxidant assay in HepG2 cells.

## 4. Conclusions

During the last decades, flaxseeds have emerged as one of the key sources of antioxidant phytochemicals. Knowledge about the variation in the accumulation of these valuable constituents is, hence, of particular interest. This study constitutes the first work devoted to the influence of genetic, edaphic, and climatic parameters on the main compounds constituting the so-called lignan macromolecule of flaxseeds, and the antioxidant activities of the obtained extracts. Our results evidenced the predominant influence of genetic factors (cultivar) on the accumulation of the constituents of the lignan macromolecule in flaxseeds. The results of gene expression suggest a transcriptional regulation of this accumulation, knowledge of which would help to manipulate the phenolic contents of flax. Elucidating the complete transcription regulation of lignan biosynthesis in flax would, therefore, help to better control their accumulation. In our hands, other environmental parameters, such as geographic and climatic variables, did not result in significant changes in the lignan macromolecule accumulation. Both in vitro and in vivo antioxidant activity relied on SDG, CafG, and CouG accumulations. Future works using purified compounds will be conducted to further elucidate their respective contribution to the cellular antioxidant capacity observed with flaxseed extracts. Considering the health benefits of these compounds, the present study evidenced the importance of a better knowledge of the flax cultivar type that could greatly influence the phytochemical intakes and the associated biological activities. Therefore, we recommend that this crucial parameter be considered in epidemiological studies dealing with flaxseeds.

## Figures and Tables

**Table 1:** Influence of the cultivar (C), cultivation site (L), and year (Y) on the accumulation of the main constituents of the lignan macromolecule in flaxseeds.

Cultivar	Location_Year	SDG ^a^	HDG ^a^	FerG ^a^	CouG ^a^	CafG ^a^
Astral	AIR_05	12.85 ± 0.14	0.98 ± 0.06	1.65 ± 0.06	5.88 ± 0.09	0.85 ± 0.04
	CHA_05	12.53 ± 0.11	1.05 ± 0.03	1.90 ± 0.08	6.05 ± 0.08	0.98 ± 0.06
	GAM_03	11.73 ± 0.06	0.88 ± 0.04	1.58 ± 0.07	4.80 ± 0.08	1.23 ± 0.06
	GAM_04	11.68 ± 0.09	1.10 ± 0.05	1.58 ± 0.04	4.78 ± 0.05	1.25 ± 0.05
	GAM_05	13.48 ± 0.13	0.85 ± 0.01	1.57 ± 0.02	6.07 ± 0.22	0.87 ± 0.02
Barbara	AIR_05	21.68 ± 0.17	0.93 ± 0.06	1.95 ± 0.03	10.48 ± 0.12	1.83 ± 0.04
	CHA_05	20.88 ± 0.07	1.05 ± 0.03	2.18 ± 0.07	8.63 ± 0.65	1.63 ± 0.04
	GAM_03	20.63 ± 0.10	0.95 ± 0.03	1.78 ± 0.03	9.95 ± 0.11	1.33 ± 0.06
	GAM_04	21.85 ± 0.34	0.85 ± 0.03	1.78 ± 0.02	9.85 ± 0.26	1.53 ± 0.06
	GAM_05	21.85 ± 0.81	0.88 ± 0.01	1.66 ± 0.01	9.84 ± 0.07	1.53 ± 0.01
Baladin	AIR_05	16.03 ± 0.11	1.15 ± 0.03	2.10 ± 0.05	7.88 ± 0.07	1.48 ± 0.07
	CHA_05	15.68 ± 0.19	1.18 ± 0.03	2.28 ± 0.07	7.58 ± 0.10	1.43 ± 0.09
	GAM_03	18.20 ± 0.38	1.03 ± 0.03	2.03 ± 0.04	7.88 ± 0.12	1.33 ± 0.02
	GAM_04	16.45 ± 0.13	1.08 ± 0.07	2.08 ± 0.02	7.33 ± 0.21	1.40 ± 0.08
	GAM_05	16.20 ± 0.38	0.95 ± 0.01	1.92 ± 0.01	6.91 ± 0.04	1.19 ± 0.01
Baïkal	AIR_05	8.33 ± 0.13	1.03 ± 0.04	1.75 ± 0.07	4.85 ± 0.07	0.85 ± 0.01
	CHA_05	8.23 ± 0.07	1.18 ± 0.02	1.75 ± 0.05	4.85 ± 0.06	1.05 ± 0.03
	GAM_03	8.23 ± 0.17	1.15 ± 0.05	1.88 ± 0.07	4.90 ± 0.07	0.80 ± 0.05
	GAM_04	8.45 ± 0.10	1.05 ± 0.08	1.98 ± 0.03	4.98 ± 0.08	0.98 ± 0.04
	GAM_05	8.40 ± 0.04	0.98 ± 0.01	1.79 ± 0.02	4.88 ± 0.05	0.83 ± 0.01
Oliver	AIR_05	21.00 ± 0.25	0.75 ± 0.03	1.55 ± 0.03	10.15 ± 0.09	1.90 ± 0.05
	CHA_05	19.50 ± 0.19	0.85 ± 0.03	1.33 ± 0.06	9.03 ± 0.26	1.75 ± 0.06
	GAM_03	19.95 ± 0.11	0.98 ± 0.04	1.18 ± 0.06	9.38 ± 0.07	1.33 ± 0.06
	GAM_04	19.93 ± 0.11	1.03 ± 0.07	1.03 ± 0.03	9.35 ± 0.09	1.45 ± 0.05
	GAM_05	21.55 ± 0.37	0.83 ± 0.01	1.06 ± 0.01	9.12 ± 0.08	1.37 ± 0.01
*F values*	Cultivar (C)	284.62 ***	4.06 *	19.18 ***	95.33 ***	14.76 ***
	Location (L)	0.02	1.21	1.04	0.13	0.61
	Year (Y)	0.03	2.09	0.68	0.02	0.48
	C × L	194.69 ***	3.57 *	21.90 ***	75.96 ***	11.94 ***
	C × Y	198.99 ***	4.58 *	17.25 ***	59.81 ***	11.19 ***
	Y × L	0.02	1.484	0.536	0.062	0.441
	C × L × Y	148.70 ***	4.23 *	16.18 ***	51.26 ***	9.62 ***

^a^ All contents are given in mg/g DW. Values are mean ± SD of 4 independent replicates. ANOVA, F represents the effect. Significance level: * *p* < 0.05; ** *p* < 0.01; *** *p* < 0.001.

**Table 2:** Correlation analysis using Pearson correlation coefficient (PCC).

Variables	SDG	HDG	FerG	CouG	CafG	FRAP	ORAC	Iron Chelation	MDA inhibition
SDG									
HDG	−0.515 **								
FerG	−0.231 ^ns^	0.510 **							
CouG	0.966 ***	−0.476 *	−0.215 ^ns^						
CafG	0.835 ***	−0.360 ^ns^	−0.063 ^ns^	0.832 ***					
FRAP	0.676 ***	−0.624 ***	−0.545 **	0.639 ***	0.671 ***				
ORAC	0.669 ***	−0.325 ^ns^	−0.170 ^ns^	0.676 ***	0.717 ***	0.573 **			
Iron Chelation	0.758 ***	−0.627 ***	−0.692 ***	0.768 ***	0.661 ***	0.817 ***	0.665 ***		
MDA inhibition	0.867 ***	−0.666 ***	−0.482 *	0.806 ***	0.721 ***	0.774 ***	0.617 ***	0.875 ***	

Significance level: * *p* < 0.05; ** *p* < 0.01; *** *p* < 0.001; ns: not significant.

**Table 3:** Influence of the cultivar (C), cultivation site (L), and year (Y) on the in vitro and in vivo antioxidant activities of flaxseed extracts.

Cultivar	Location_Year	FRAP ^a^	ORAC ^a^	Iron Chelation ^b^	MDA Inhibition ^c^
Astral	AIR_05	252.55 ± 2.45	281.55 ± 11.16	9.57 ± 0.25	37.10 ± 0.28
	CHA_05	264.41 ± 10.28	317.87 ± 8.00	10.11 ± 0.41	36.95 ± 0.43
	GAM_03	322.81 ± 1.41	263.13 ± 11.91	9.66 ± 0.12	38.47 ± 0.75
	GAM_04	276.68 ± 4.33	312.34 ± 1.12	9.31 ± 0.53	37.71 ± 1.18
	GAM_05	252.55 ± 5.23	263.92 ± 4.56	9.57 ± 0.22	38.17 ± 1.07
Barbara	AIR_05	332.55 ± 4.57	339.45 ± 1.95	11.97 ± 0.19	45.95 ± 2.26
	CHA_05	317.61 ± 9.33	341.29 ± 16.09	11.35 ± 0.72	44.58 ± 1.08
	GAM_03	292.81 ± 5.84	329.45 ± 8.65	11.79 ± 0.06	43.05 ± 3.23
	GAM_04	296.41 ± 8.20	336.55 ± 2.14	12.32 ± 0.53	46.41 ± 0.43
	GAM_05	331.48 ± 5.23	309.45 ± 3.72	10.99 ± 0.28	45.65 ± 1.94
Baladin	AIR_05	217.35 ± 4.67	286.82 ± 2.79	8.69 ± 0.31	33.44 ± 2.16
	CHA_05	240.55 ± 6.31	334.18 ± 5.95	8.24 ± 0.44	33.28 ± 0.97
	GAM_03	254.01 ± 2.30	307.61 ± 4.18	8.16 ± 0.43	32.06 ± 0.64
	GAM_04	288.41 ± 11.27	321.82 ± 2.60	8.87 ± 0.06	28.85 ± 0.43
	GAM_05	255.08 ± 14.24	281.03 ± 2.70	7.80 ± 0.59	34.50 ± 2.16
Baïkal	AIR_05	262.01 ± 3.21	278.66 ± 5.76	8.42 ± 0.37	25.34 ± 1.18
	CHA_05	274.95 ± 3.49	281.03 ± 2.79	8.16 ± 0.40	24.89 ± 1.19
	GAM_03	227.61 ± 1.41	305.24 ± 2.32	7.18 ± 0.56	23.66 ± 1.62
	GAM_04	243.75 ± 5.42	269.97 ± 4.09	7.62 ± 0.55	21.68 ± 3.13
	GAM_05	243.78 ± 1.27	286.29 ± 2.51	8.07 ± 0.12	26.11 ± 0.86
Oliver	AIR_05	350.68 ± 0.81	368.66 ± 21.39	14.45 ± 0.25	47.02 ± 2.16
	CHA_05	306.28 ± 5.27	374.45 ± 6.42	14.54 ± 0.44	46.87 ± 1.19
	GAM_03	302.41 ± 6.17	278.92 ± 5.39	13.74 ± 0.38	43.21 ± 0.75
	GAM_04	355.75 ± 8.20	326.55 ± 2.70	14.72 ± 0.47	45.34 ± 2.37
	GAM_05	343.08 ± 5.28	375.76 ± 7.91	14.10 ± 0.38	46.87 ± 1.40
*F values*	Genetic (C)	11.91 ***	5.37 ***	188.00 ***	161.33 ***
	Location (L)	0.02	1.05	0.04	0.03
	Year (Y)	0.06	0.15	0.04	0.08
	C × L	7.20 **	4.59 **	133.03 ***	105.31 ***
	C × Y	7.34 **	3.40 *	133.16 ***	130.60 ***
	Y × L	0.03	0.64	0.04	0.06
	C × L × Y	4.91 *	3.41 *	114.80 ***	109.27 ***

^a^ expressed in mM of Trolox C equivalent antioxidant capacity (TEAC); ^b^ expressed in µM of fixed Fe^2+^; ^c^ expressed in % inhibition of MDA formation relative to control cells; values are mean ± SD of 4 independent replicates. ANOVA, F represents the effect. Significance level: * *p* < 0.05; ** *p* < 0.01; *** *p* < 0.001.

## Supplementary Materials

The following are available online, Table S1: Meteorological characteristics of the cultivation site; Figure S1: Scheme describing cultivation conditions; Figure S2: Climatic data for the trial sites Airaines (AIR), Gamaches-en-Vexin (GAM) and Chartres (CHA) for the years 2003 (03), 2004 (04), and 2005 (05). Precipitations are expressed as cumulative monthly rainfall in mm, and temperatures are an average of daily temperature in °C.
