# Supplementary material for: Insight into the Influence of Cultivar Type, Cultivation Year, and Site on the Lignans and Related Phenolic Profiles, and the Health-Promoting Antioxidant Potential of Flax (Linum usitatissimum L.) Seeds
Source: Molecules. 2018 Oct 14;23(10):2636. doi: 10.3390/molecules23102636 (PMC6222607; doi:10.3390/molecules23102636)
Supplement: Supplementary file 1 [file molecules-23-02636-s001.pdf]

molecules

Supplementary Materials

Insight into the influence of cultivar type, cultivation year and site on the lignans and related phenolic profiles and the health promoting antioxidant potential of flax (*Linum usitatissimum* L.) seeds

Laurine Garros[1,2,3,§], Samantha Drouet[1,2,§], Cyrielle Corbin[1,2], Cédric Decourtil[1,2], Thibaud Fidel[1,2], Julie Lebas de Lacour[1,2], Emilie A. Leclerc[1,2], Sullivan Renouard[1,2], Duangjai Tungmunnithum[1,2,4], Joël Doussot [1,2,5], Bilal Haider Abassi[1,2,6], Benoit Maunit[2,3], Éric Lainé[1,2], Ophélie Fliniaux[7], François Mesnard[7], Christophe Hano[1,2,*]

[1] Laboratoire de Biologie des Ligneux et des Grandes Cultures (LBLGC) EA1207 INRA USC1328, Plant LIGNANS Team, Université d'Orléans, 28000 Chartres, France ; laurine.garros@univ-orleans.fr; samantha.drouet@univ-orleans.fr; cyrielle.corbin@univ-orleans.fr; cedric.decourtil@univ-orleans.fr; thibaud.fidel@univ-orleans.fr; julie.lebas-de-lacour@univ-orleans.fr; emilie.leclerc@univ-orleans.fr; sullivan.renouard@univ-orleans.fr; eric.laine@univ-orleans.fr; hano@univ-orleans.fr

[2] COSM'ACTIFS, Bioactifs et Cosmétiques, CNRS GDR3711, 45067 Orléans Cedex 2, France

[3] Institut de Chimie Organique et Analytique (ICOA) UMR7311, Université d'Orléans-CNRS, 45067 Orléans Cedex 2, France ; benoit.maunit@univ-orleans.fr

[4] Department of Pharmaceutical Botany, Faculty of Pharmacy, Mahidol University, 447 Sri-Ayuthaya Road, Rajathevi, Bangkok 10400, Thailand; duangjai.tun@mahidol.ac.th

[5] Le CNAM, Ecole Sciences Industrielles et Technologies de l'Information (SITI), Chimie Alimentation Santé Environnement Risque (CASER), Paris, France ; joel.doussot@lecnam.net

[6] Department of Biotechnology, Quaid-i-Azam University, 45320 Islamabad, Pakistan; bhabbasi@qau.edu.pk

[7] Biologie des Plantes et Innovation (BIOPI) EA 3900, Université de Picardie Jules Verne, 80000 Amiens, France ; ophelie.fliniaux@u-picardie.fr; francois.mesnard@u-picardie.fr

§ These two authors contributed equally to this work and should be considered both as first authors

* Correspondence: hano@univ-orleans.fr; Tel.: +33-237-309-753

Table S1 Meteorological characteristics of the cultivation site

			TN	TX	TNN	TXX	HRR	RMAX	INST
**September**	**AIR**	**2004**	12,4	20,4	6,2	28	36	7	181
	**CHA**	**2004**	11,3	22,7	3	31,3	11	6,8	210
	**GAM**	**2002**	9,4	20	1,9	23,8	10,6	4,2	169
		**2003**	9,7	22	2,9	30,3	23,2	16,4	217
		**2004**	11,8	21,7	6,1	29,5	17,8	10	186
**October**	**AIR**	**2004**	9,1	15,5	5,2	20,5	49,6	10,8	101
	**CHA**	**2004**	8,2	16,1	2,9	24,5	102,2	16,6	98
	**GAM**	**2002**	7,5	15,9	1,4	22,1	102,8	29,8	111
		**2003**	5,6	13	-3,1	24,2	64,8	16,2	106
		**2004**	8,6	15,7	3,5	22,6	53,8	12,2	93
**November**	**AIR**	**2004**	5,1	10,3	-1	14,5	53,6	12,8	64
	**CHA**	**2004**	4,8	10	-2,1	14,1	44,6	12,2	61
	**GAM**	**2002**	6	11,7	1,4	16,3	99,4	20,6	60
		**2003**	5,5	11,4	-0,4	16,4	58,2	13,6	62
		**2004**	4,7	10	-2,7	13	45,2	9	46
**December**	**AIR**	**2004**	1,6	6,2	-3,5	11,2	76,8	28,2	45
	**CHA**	**2004**	0,7	5,5	-5,4	11,3	63,6	13	42
	**GAM**	**2002**	4,3	8,4	-2,9	14,2	70,8	14	23
		**2003**	2,3	7,2	-5,9	13,3	47,8	11,8	68
		**2004**	0,8	5,9	-4,7	11,7	62,8	19	45
**January**	**AIR**	**2005**	3,3	8,1	-4,3	13,7	40,6	11,2	95
	**CHA**	**2005**	2,5	7,5	-3,9	11,7	55,6	19,4	72
	**GAM**	**2003**	-0,1	5,4	-9,2	15,1	68,4	13,6	71
		**2004**	2,5	7,5	-4,9	14,2	84	15,8	48
		**2005**	2,4	7,9	-3,4	12,1	55	20,2	76
**February**	**AIR**	**2005**	0,7	5,5	-9,4	11,9	45,8	12,4	85
	**CHA**	**2005**	0	5,9	-7,9	12,8	23,4	4	79
	**GAM**	**2003**	0,2	6,8	-6,4	14,5	24,2	8,2	106
		**2004**	1,8	8,1	-5,7	16,6	11	4,4	87
		**2005**	0,5	5,6	-6,7	12	14	2,6	64
**March**	**AIR**	**2005**	3,6	10,3	-9,8	22,3	31,2	14,2	95
	**CHA**	**2005**	2,8	11,4	-11	22,3	46	14,8	123
	**GAM**	**2003**	4,5	14,4	-1,2	21,4	17,2	9	185
		**2004**	2,4	10,4	-5,4	21,7	42,4	9,6	152
		**2005**	3	11,2	-7,8	22	25,8	8,8	108

**April**	**AIR**	**2005**	6,5	14,2	0,4	23,4	47,4	11	169
	**CHA**	**2005**	5,5	15,4	-0,6	26,8	51,4	14,6	157
	**GAM**	**2003**	5,3	16	-3,3	24,2	27,4	11,6	197
		**2004**	5,4	14,4	0	20,9	76	14,6	149
		**2005**	5,7	15	0,1	25,7	49	9	151
**May**	**AIR**	**2005**	8,5	16,6	1,2	31,4	84	22	212
	**CHA**	**2005**	8,5	18,8	1,8	30,8	69,2	16,2	210
	**GAM**	**2003**	8,4	18,3	1,7	28,2	43,2	14,6	191
		**2004**	7	17,3	1,5	24	36,8	9,8	245
		**2005**	8,3	17,5	1,2	30	60,8	25	194
**June**	**AIR**	**2005**	11,5	21,9	4,7	32,1	29,8	10,2	255
	**CHA**	**2005**	12,1	24,5	5,4	33,9	67,6	17	274
	**GAM**	**2003**	14,1	24,6	9,1	31,3	45,8	16,6	225
		**2004**	11,3	22	4,1	30,2	14,2	3,2	225
		**2005**	12	23,1	5,3	32,4	35,2	9,6	240
**July**	**AIR**	**2005**	13,7	21,6	10,5	28,5	133,6	49	184
	**CHA**	**2005**	13,9	24,6	8,4	33,3	48,8	10,2	195
	**GAM**	**2003**	14,1	24,7	9,1	34,4	43,4	17	221
		**2004**	12,8	22,6	6,6	29,1	65,8	17,2	197
		**2005**	13,7	23,3	7,6	30,2	75,6	34,4	nd
**August**	**AIR**	**2005**	12	21,4	7,6	32,5	61,4	14,8	240
	**CHA**	**2005**	11,5	24,5	6,5	33	25,6	10	253
	**GAM**	**2003**	15,6	28,7	7,7	38,4	5,8	1,8	269
		**2004**	14,5	24	8,3	32,2	69,8	13,6	168
		**2005**	11,6	23,2	6,7	33	23,4	8,2	nd

TN: Mean maximum temperature (day) in °C; TX: Mean minimum temperature (night) in °C; TXX: Absolute maximum temperature in °C; TNN: Absolute minimum temperature in °C; HRR: cumulative precipitation in mm; RMAX: Maximal precipitation for a 24h-period in mm; INST: cumulative sunshine duration in h; data collected from Meteo France stations

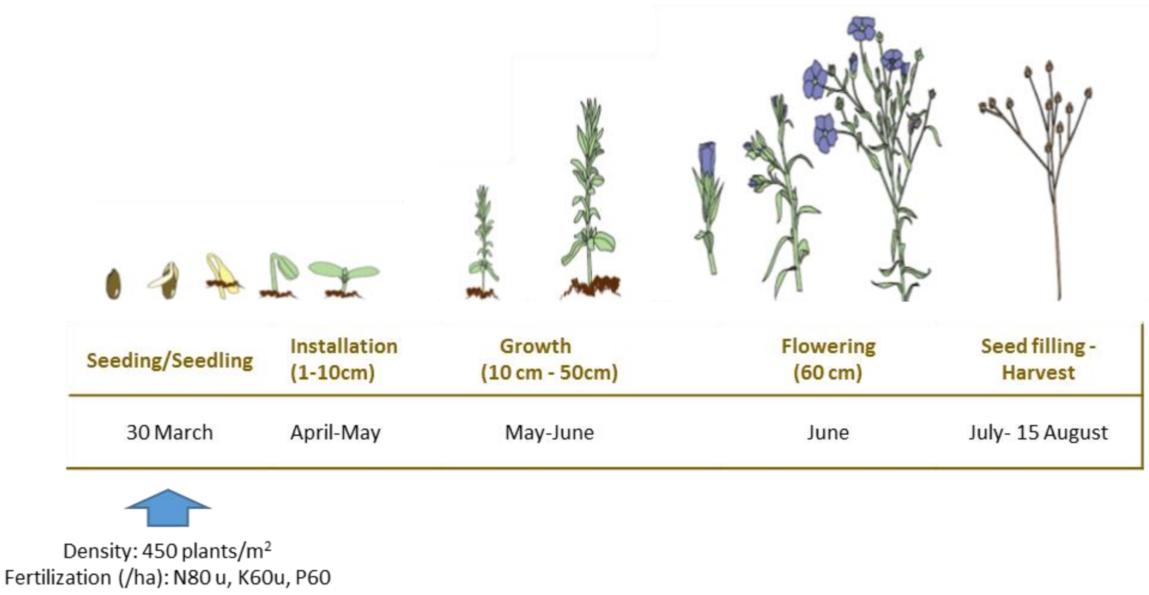

Figure S1: Scheme describing the flax growth and cultivation cycle

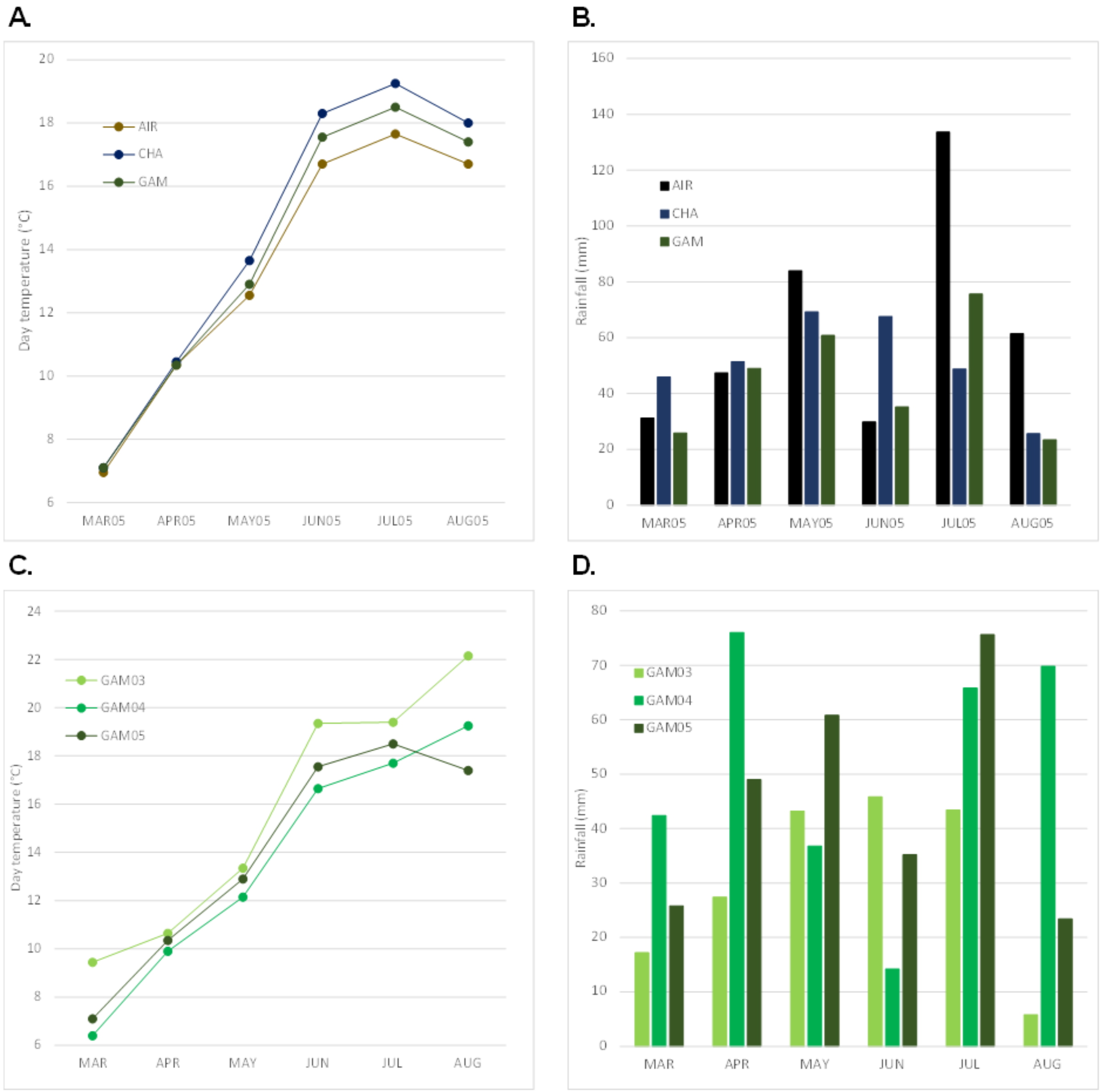

Figure S2: Climatic data for the trial sites Airaines (AIR), Gamaches-en-Vexin (GAM) and Chartres (CHA) for the years 2003 (03), 2004 (04) and 2005 (05). Precipitations are expressed as cumulative monthly rainfall in mm and temperatures are average of daily temperatures in °C.
